# Optimizing COP by RSM and MATLAB model of mini refrigerator based on thermoelectric units driven by solar photovoltaic

**DOI:** 10.1038/s41598-024-72500-1

**Published:** 2024-10-01

**Authors:** Amal E. M. Elnaggar, Soliman Sharaf, Zeinab S. Abedel Rehim, M. A. El-Bayoumi, Hassan M. M. Mustafa, Helmy M. El Zoghby

**Affiliations:** 1 National Research Centre, Mechanical Engineering Department, Engineering and Renewable Energy Research Institute, Giza, Egypt; 2 Electrical Power and Machines Engineering Department, Faculty of Engineering, Helwan University, Helwan, Egypt

**Keywords:** Thermoelectric unit, Controller, MATLAB Simulink, RSM, Solar photovoltaic, Renewable energy, Energy science and technology, Engineering

## Abstract

Energy scarcity in the world and the pollutants resulting from excessive use of conventional energy aroused the need for sustainable alternatives that are environment friendly. A multi-use thermoelectric refrigerator powered by solar energy to obtain the lowest consumption with the highest efficiency. The designed refrigerator is based on the Peltier effect using Peltier units where a temperature difference is created between the junctions by applying a voltage difference across the junction. This study investigates the performance of a refrigerator cooling system powered by a photovoltaic (PV) system. The research aims to assess the efficiency, effectiveness, and feasibility of utilizing solar energy to drive refrigeration, particularly in off-grid or environmentally conscious applications. Through a comprehensive experimental setup and data analysis, the study examines energy consumption, cooling efficiency, and overall system performance under varying conditions. The findings contribute valuable insights into the potential of PV-powered refrigerators as sustainable cooling solutions. It relies on a control unit that measures the resulting temperature to determine the appropriate connection mode to give the highest cooling efficiency. The average solar radiation when operating for 8 h, for the different seasons of the year was 149.5, 67.5, 119.3, and 118.3 w/m^2^ in summer, winter, spring, and fall, respectively. The average cooling energy consumption was 107.25, 137.04, 107, and 138.08 w for temperatures (20 ± 1, 15 ± 1, 20 ± 2, and 15 ± 2) °C respectively that proof solar radiation is sufficient to produce energy for the summer of cooling temperatures up to 15 °C, while in the spring and fall it is sufficient to 20 °C. The Fast not Eco mode is the least energy consuming and the fastest cooling, it can be used for rapid cooling at a short time less than an hour. The best mode in the case of continuous operation is the case of as next Eco mode cooling temperature of 20 ± 0.1 °C. The MATLAB Simulink model was developed to reduce the design cycle and facilitate the integration of solar photovoltaic with the TEC. The optimal operating point is identified through simulation and validated through experimental analysis, the optimal COP was 71.089% by Response surface methodology (RSM).

## Introduction

In recent years, thermoelectric devices have gained widespread use in daily life due to their practical, efficient, and environmentally friendly design. There are two main types of thermoelectric devices: thermoelectric generators (TEGs) and thermoelectric coolers (TECs).

Thermoelectric devices serve dual purposes: they can either harness cooling power through electrical energy or generate electrical energy from temperature differentials^1^. These devices are primarily categorized into thermoelectric generators (TEGs) and coolers (TECs). TEGs leverage the Seebeck effect to convert temperature differences across two surfaces into electrical energy. Conversely, TECs operate based on the Peltier effect^2,3^.

In a TEC setup, when two semiconductor materials (one of type P and the other of type N) are electrically connected in series and thermally connected in parallel, applying a current induces one surface to cool and the other to heat, thereby manifesting the Peltier effect,^5,6^.

Compared to traditional cooling systems, thermoelectric coolers (TECs) offer several significant advantages. They do not contain moving parts or fluids that can harm the environment, operate silently and without vibrations, provide precise temperature control, incur no maintenance costs, and feature a compact structure^7^. These attributes make TECs highly versatile, finding applications across a broad spectrum from space and military industries to wearable technologies^8^. There is a substantial body of research on thermoelectric (TE) cooling that combines theoretical and experimental data. To create more effective heat exchangers, typical shell and tube heat exchangers have been modified to use a tubular TE device composed of tilted multilayers of BST/Ni as the tubes^9–11^.

Despite their advantages, thermoelectric coolers (TECs) still face challenges with low coefficient of performance (COP) values compared to traditional refrigeration technologies^12^. This limitation has spurred researchers to explore new methods to enhance efficiency.

A computational study was conducted on a thermoelectric refrigerator with an interior volume of 15 cubic meters to examine how the heat exchanger affects the refrigerator's overall energy consumption and efficiency. The findings indicated that significant improvements in thermoelectric cooling efficiency can be achieved through proper optimization of the heat exchangers^13^.

One approach involves incorporating liquid-cooled blocks to improve system efficiency by enhancing heat removal from the hot side of the TEC^14^. Typically, these systems utilize pure water as a working fluid. However, in recent years, researchers have turned to Nano fluids as an alternative working fluid. Nano fluids are suspensions of nanoparticles (such as metal oxides or carbon nanotubes) in conventional fluids like water, ethylene glycol, or kerosene. They exhibit significantly higher thermal conductivities compared to pure fluids, which can potentially improve the overall performance of thermoelectric cooling systems^15,16^. Cuce et al. study the effects of various hybrid Nano fluids on basic performance parameters such as cooling power and COP in thermoelectric cooling (TEC) applications were experimentally investigated. A cooled cabinet with an interior volume of 36 L was designed and manufactured^17^.

Abdulghani showed endeavors are made to provide an experimental-based optimization process for the Peltier air cooler using the well-known Taguchi method Studies show that, for a given total input power, more modules produce a better coefficient of performance (COP). The Peltier cooler’s cost per cooling unit is reduced by using the optimal number of modules,^18^. Kherkhar et al. employ both experimental and numerical methods to assess the performance and efficiency of a thermoelectric cooler (TEC) control system. The refrigeration system is designed using semiconductor materials operating under the Peltier effect, and an Arduino device^19^. Afshari proved that the average COP value of the air-to-water mode is approximately 30–50% higher than that of the air-to-air mode. In other words, an air-to-water thermoelectric cooling device operates more efficiently^20^.

Chen et al. presented a model of an internal and external irreversible thermoelectric generator-driven thermoelectric refrigerator. They found that the coefficient of performance (COP) is no longer constant and declines monotonically with an increase in the total number of thermoelectric devices. Additionally, the cooling load decreases and is no longer proportionate to the total number of thermoelectric units^21^. In the same field of research, A. Çağlar designed a portable thermoelectric (TE) refrigerator. The temperature and COP of the TE refrigerator were examined for optimal operational settings over 60 min using the orthogonal fractional factorial experiment design approach. Results revealed that although the COP decreased from 0.351 to 0.011, the air temperature inside the TE refrigerator dropped from 293 to 254.8 K^22^. Irshad et al. provide a comprehensive review of photovoltaic (PV) and thermoelectric (TE) technologies in the context of energy-efficient building development. It discusses and critically analyzes their implementations. The basics of solar panels and their performance parameters are introduced, followed by an exploration of integrating PV technology into buildings^23^.

Kaiprath and Kishor Kumar investigated thermoelectric (TE) refrigeration systems, based on the Peltier effect; offer an alternative to traditional systems. They're noise-free, and vibration-free, and use electrons as heat carriers instead of refrigerants. Their biggest advantage that They can run directly on solar PVs, which provide DC power^24^. Studying the performance of the electrical network and mitigating the environmental impact. This study seeks to evaluate the performance of a PV-powered refrigerator cooling system, addressing key questions regarding energy efficiency, reliability, and practicality^25^. Singh et al. Study the explore correlation between solar module efficiency and the impact of thermoelectric cooling in conjunction with phase change material PCM. The thermoelectric effect, attributed to the “Peltier effect,” and the cooling effect of PCM, based on latent energy storage, are combined to enhance solar cell performance^26^.

Response surface methodology (RSM) represents a statistical technique employed to design, analyze, and refine experiments within any given cycle. Among the various types of plans, the central composite design stands out as a frequently utilized tool in the literature for enhancing and optimizing diverse processes^27^. In statistics, RSM is thought to be a very useful tool for studying the relationship between input factors and output using models and how factorial variables affect the response. Utilizing the “one variable, once” procedure to enhance the boundaries needs countless trials, though RSM can achieve this with altogether fewer runs. Besides, the "one factor at a time" procedure can't expect the consolidated impact of at least two variables on the result, which is conceivable utilizing RSM^28^.

The primary objective of this research is to study the efficiency of a thermoelectric refrigerator under various operational settings. The goal is to meet the daily needs of residents of remote areas by using renewable energy sources without the need to store energy. Different configurations of Peltier elements, along with different voltages generated by the photovoltaic cells, will be tested to control target temperatures. By achieving this, a sustainable system can be created, which indirectly contributes to reducing energy production emissions.

## Materials and method

### Refrigerator box

**Fig. 1:**
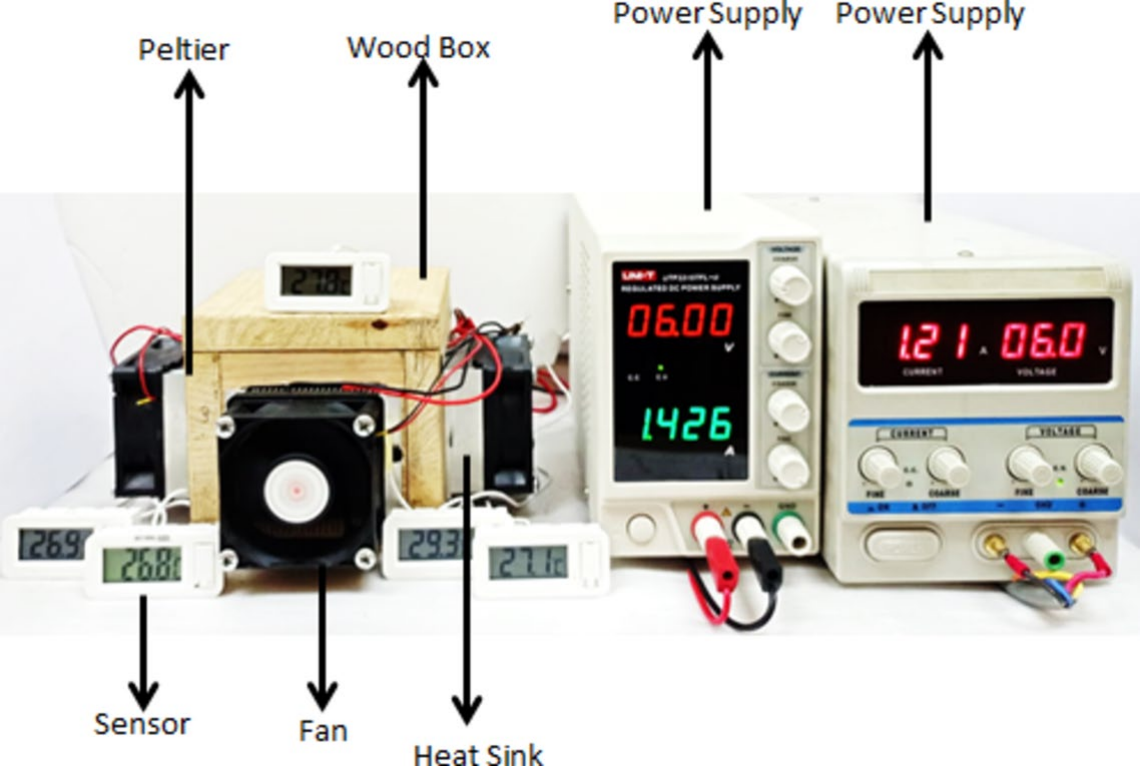
Photographic picture of refrigerator box.

Figure 1 shows a photograph of the portable refrigerator. The setup consists of a refrigerator box (RB) measuring 8 cm × 8 cm × 8 cm, the RB volume is 0.000512 m^3^ (512 cm^3^) with four thermoelectric units (TUs) placed on its walls thermoelectric units (TU); placed on the walls of the four sides of the refrigerator box^29^. The RB consists of two boxes: an outer wooden box and an inner stainless-steel box^30^ separated by thermal insulation foam to prevent cooling loss. Each TU includes a Peltier unit, heat sinks, and a fan. Effective cooling on the cold side of the Peltier unit requires effective cooling of its hot side, which is typically facilitated by a fan. Measurements are made using a digital multi-meter for voltage and current, while the temperature is monitored with a digital thermometer.

### Material and characteristics of experimental components

**Fig. 2:**
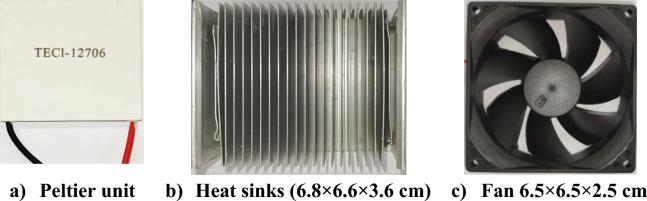
Peltier unit, Heat sinks and Fan.

The cooling system incorporates a Peltier unit, specifically the TEC1-12706 model^31^, which utilizes N-P junction semiconductors soldered to a copper conductor (Fig. 2a). This unit is complemented by an aluminum heat sink with fins (Fig. 2b) and a fan measuring 6.5 cm × 6.5 cm × 2.5 cm (Fig. 2c). Detailed performance specifications for the TEC1-12706 Peltier unit are listed in Table 1**.**

**Table 1:** Performance specifications for the TEC1-12706 Peltier unit.

Model	length, (m)	Power, Q_max_, (W)	Current, I_max_, (A)	Voltage, V_max_, (V)
TEC1-12706	0.04	50	6.4	14.4

### PV systems in refrigeration applications

**Fig. 3:**
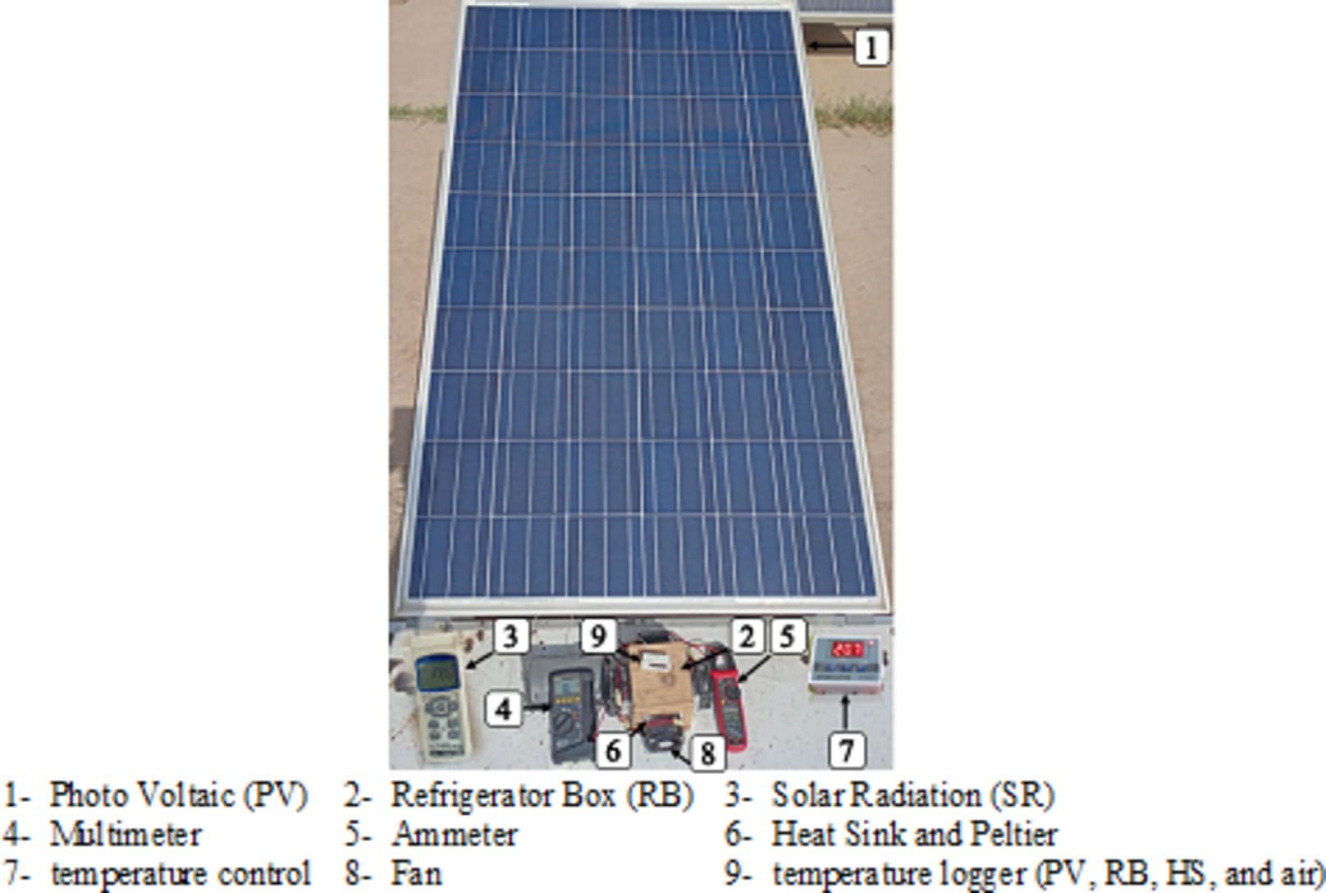
Photographic picture of refrigerator box with PV module.

Advances in photovoltaic systems have made it practical to integrate PV technology to offset energy consumption for refrigeration. Despite receiving 1000 W/m^2^ of solar irradiance on a sunny day, the electrical power extracted from a panel can be relatively modest. For instance, a 4 m^2^ panel with 25% efficiency can yield up to 1 kW under clear sky conditions at noon. However, typical refrigeration systems in tropical regions require more electric power (ranging from 300 to 2000 W) than a single panel can provide, even during peak sunlight hours. Figure 3 illustrates a refrigerator box connected to a PV module and control system. Figure 3 displays the refrigerator box connected with photo voltaic system the Solar Thermoelectric Refrigerator (STER) consists of three main components: a refrigerator box attached to the Peltier unit to facilitate efficient heat dissipation, PV module, and control unit. The performance of the STER was evaluated under hot weather conditions from August to October 2023 in Giza, Egypt, where the average outside temperatures ranged from 25 to 55 °C. Throughout the study, the temperature inside the refrigerator box was monitored continuously from sunrise to sunset.

### Control unit

In our setup, we employed two control systems: the XH-W3002 digital temperature controller to maintain temperatures inside the box at precise levels (20 ± 0.1 °C, 15 ± 0.1 °C, 10 ± 0.1 °C, 5 ± 0.1 °C), and an Arduino (UNO Rev3) board to manage cooling modes. The Arduino board categorizes cooling into five distinct modes:

1. Economic mode: Activated with one Peltier unit at four volts.
2. Minimum mode: Requires two Peltier units at four volts.
3. Medium mode: Utilizes four Peltier units at four volts, providing a balance between economy and speed.
4. Fast mode: Uses four Peltier units at six volts for rapid cooling when necessary.
5. Fast not economical mode: Utilizes four Peltier units at ten volts for maximum cooling power.

These modes allow for flexible control over cooling intensity based on specific operational needs.

### MATLAB Simulink simulation setup

#### Peltier MATLAB Simulink

**Fig. 4:**
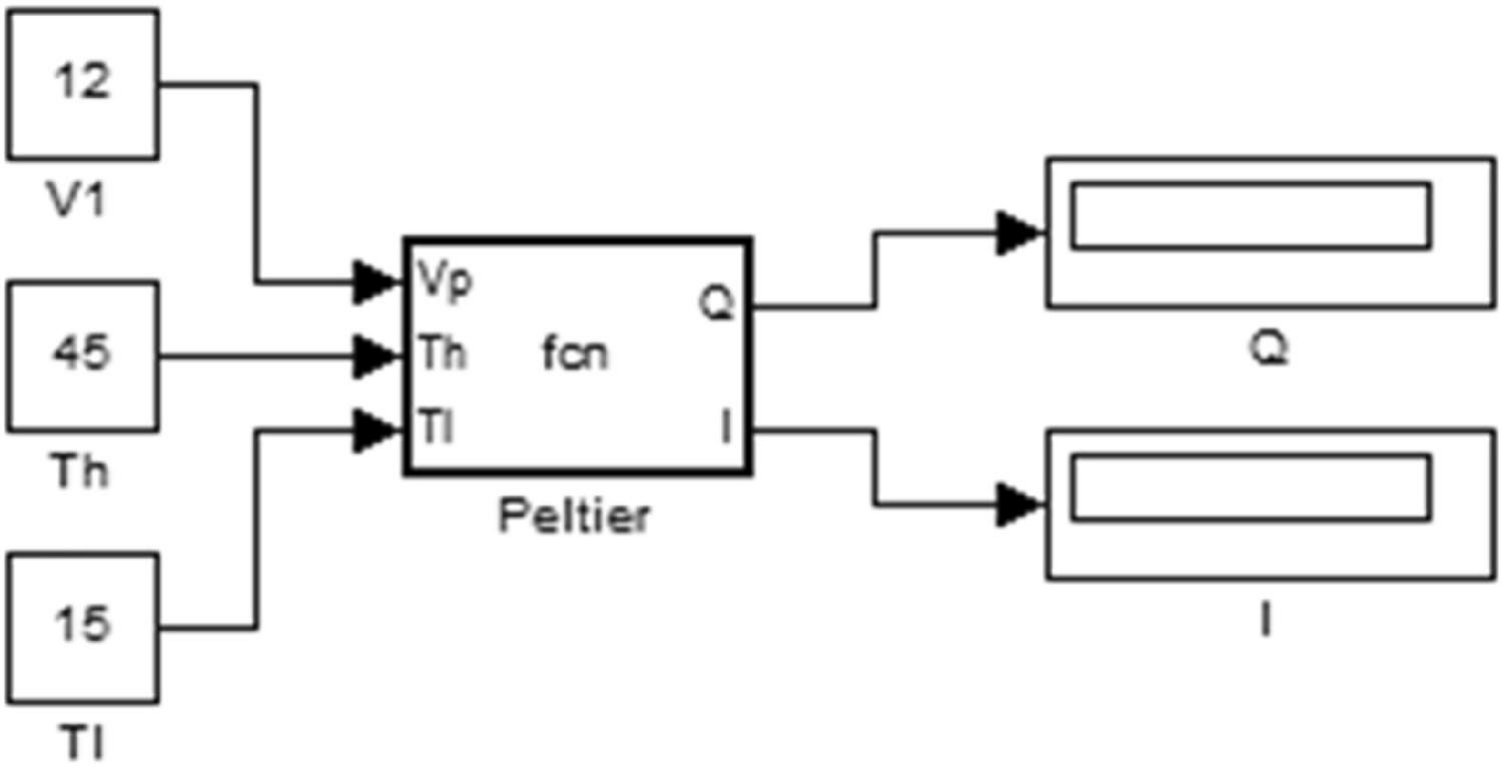
Peltier module.

The TEC model, specifically the TEC1-12706 thermoelectric cooler, is utilized to cool the plates attached to the refrigerator box. This model characterizes the relationship between temperature difference and voltage at T_h_ = 25 °C across various currents: I = 1.5, 3.0, 4.5, and 6.0 A. Additionally, a separate thermometer thermal model is applied to manage the TEC cooler. Figure 4 illustrates the Peltier module in MATLAB software version R2024a (https://www.mathworks.com/products/new_products/latest_features.html); the TEC model is integrated under a toggle controller phase. This toggle controller initiates corrective actions based on the balanced temperature of the thermoelectric cooler, synchronized with a pulse. The model incorporates thermal resistance and capacitance to effectively couple and manage the cooling system, the code 1 of MATLAB model was added in supplementary information (Supplementary file 1).

#### PV MATLAB Simulink

**Fig. 5:**
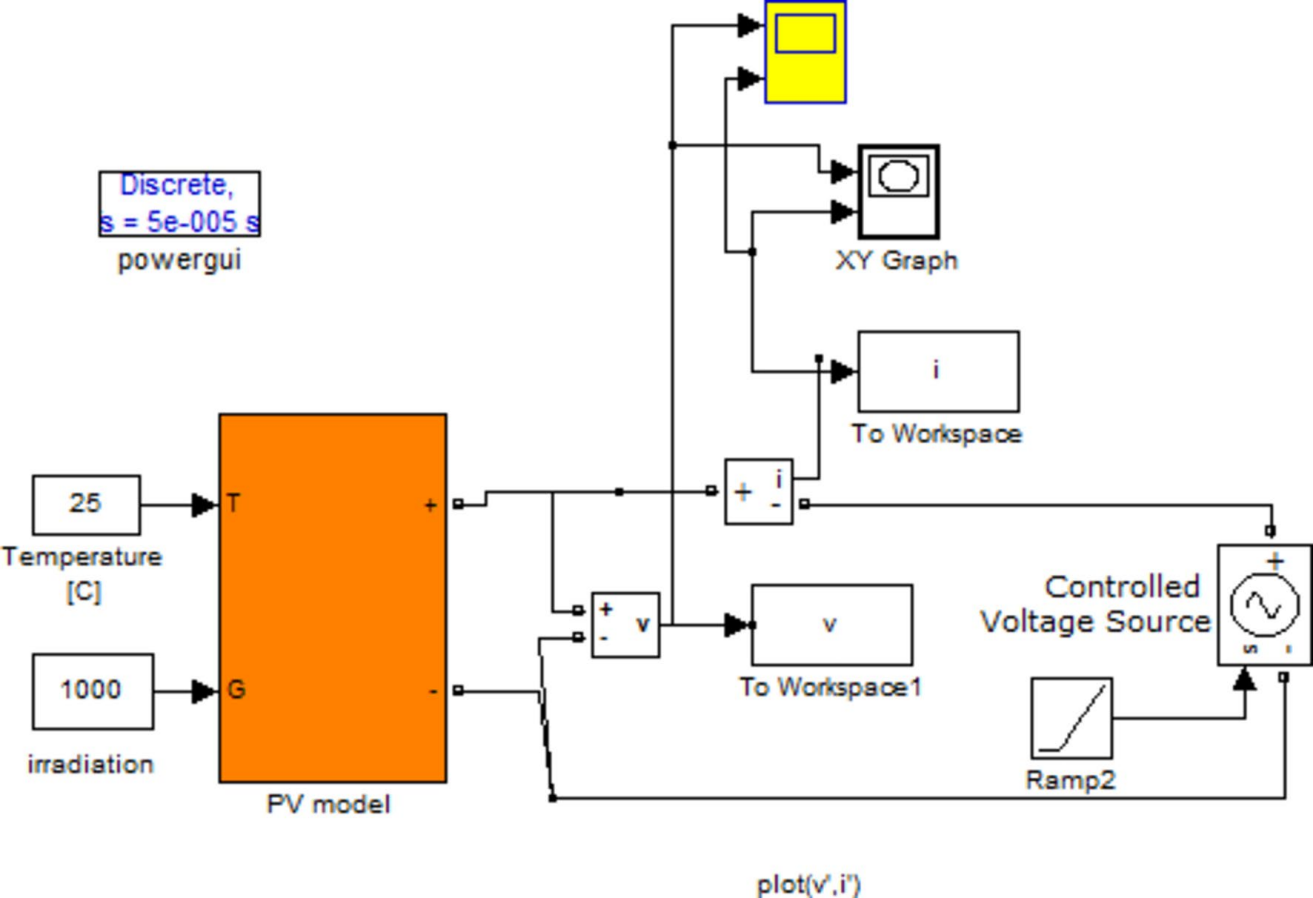
MATLAB Simulink model for constructing PV characteristics.

The software tool available here for download is developed starting with a simple MATLAB function that simulates a solar cell, Fig. 5 shows a MATLAB Simulink model for constructing PV characteristics.

The initial step in modeling involves analyzing and simulating a single photovoltaic (PV) panel to generate I–V (current–voltage) and P–V (power–voltage) curves, focusing on the maximum power point (MPP) for performance assessment. This modeling was conducted using both Simulink and Lab VIEW, based on the one-diode with serial resistance PV model, chosen for its balanced accuracy-to-complexity ratio. The specific PV panel under examination is the Sunset PX series.

### Method

#### Experimental procedure

The TE refrigerator setup includes a Peltier unit, a stainless steel (S.S.), box volume is 0.000512 m^3^ (512 cm^3^ by length 8 cm), aluminum heat sinks (plate fins), and fans. The heat sinks and fans are attached to the Peltier module to aid in effective heat dissipation. To achieve lower temperatures on the cold side of the Peltier module, the hot side must be adequately cooled. The current stabilizes and remains constant once the fans are set to a specific voltage. Meanwhile, the current drawn by the Peltier unit decreases initially during the experiment until it reaches a stable state.

Before commencing the experiments, 0.25 L of water are placed in the box, and its temperature is recorded. The experiments involve setting the voltage from 4 to 14 V, recording the ambient temperature, and measuring the current drawn by the Peltier unit and fans. Measurements of the water temperature, the hot side of the Peltier module, and current are taken every 5 min throughout the 45-min experiment duration. Multiple experiments were conducted varying the voltage (4 to 14 V), number of Peltier units (1 to 4), and initial temperature conditions.

#### Modeling study

The Peltier effect is used to design and build a TE refrigerator in this study. Peltier unit variable voltage, current, and ambient temperatures are the components of the design. According to reference^30,31^ The COP of the refrigerator system is the ratio of heat produced by the Peltier unit to energy supplied. The equations used to get the COP of the refrigerator system are the following:

1 $$\Delta \text{T}={\text{T}}_{\text{i}}-{\text{T}}_{\text{c}}$$

2 $${\text{Q}}_{\text{cooling}}={\text{mC}}_{\text{p}}\Delta \text{T}$$

3 $$\text{W}=\text{V}\times \text{I X T}$$

4 $$\text{COP}=\frac{{\text{Q}}_{\text{cooling}}}{\text{W}}$$

Q _cooling_ is the amount of heat removed from the refrigerator box (in joules).

W is the input work or energy consumed by the refrigerator box (in joules).

Cp is the specific heat of water (J/kg °C).

ΔT is the temperature change, which is the difference between the initial and final temperatures.

T _i_ is the initial temperature (°C), T_c_ is the final temperature (°C).

T is the time taken to reach from the initial temperature to the final temperature (in hours).

V is the voltage (in volts), I is the current (in amperes).

#### COP *modeling and optimization* for selected modes

The voltage of the Peltier element and the ambient temperature significantly influences the system's COP. The input parameters and output results for the thermoelectric refrigerator at the selected modes are shown in Table 2. Box-Behnken design, a powerful tool from the Response Surface Methodology (RSM) in Design Expert software version 13 (https://www.statease.com/docs/v13/) was used in this work. This design enabled us to model and study the effect of two critical factors voltage, and numbers of Peltier on COP. A comprehensive statistical analysis of variance (ANOVA) was performed to evaluate the importance of each factor and its interactions with the COP.

**Table 2:** Input parameters and output results for the thermoelectric refrigerator at the selected modes.

No	Parameters*			Results*			
	V (V)	No. of Peltier	Time (min)	I (A)	∆T (ºC)	COP (%)	Mode
1	6	1	45	1.387	8.3	38.8	
2	4	1	45	1.006	8	77.3	Eco
3	8	1	45	1.773	8.7	23.9	
4	14	1	45	3.237	9.1	7.8	
6	10	1	45	2.222	9.2	16.1	
7	12	1	45	2.668	11.2	13.6	
8	4	2	45	1.833	12.5	66.3	Min
5	6	2	45	2.518	13.7	35.3	
9	4	3	45	2.663	15.7	57.3	
10	8	2	45	3.815	16.2	20.6	
11	10	2	45	4.596	16.9	14.3	
12	6	3	45	3.854	17.6	29.6	
13	14	2	45	6.478	17.9	7.7	
14	4	4	45	3.445	18.2	51.4	Med
15	12	2	45	5.548	18.2	10.6	
16	12	3	45	8.184	18.7	7.4	
17	14	3	45	9.397	19.3	5.7	
18	10	3	45	6.849	20.4	11.6	
19	8	3	45	5.568	20.7	18.1	
20	14	4	45	11.729	22.4	5.3	
21	6	4	45	5.251	23	28.4	Fast
22	12	4	45	10.771	24.2	7.3	
23	8	4	45	7.192	25.5	17.2	
24	10	4	45	9.149	26.4	11.2	Fast not Eco

*Where (V) is the overall voltage for the Peltier and the fan, (I) the average current, and T represents the temperature differential.

From Table 2, optimal cooling is obtained by utilizing four Peltier units, 10 V, ambient temperature of 30.9 °C, and the temperature difference of the water is 26.4 °C, which is considered the best cooling. The lowest COP is 11.2%, while the highest COP is 77.3% obtained by using one Peltier at 4 V. The different mode is classified according to maximize COP, improve cooling and energy consumption, and achieve the quickest cooling time with the lowest temperature.

## Results and discussion

Several experiments were conducted using varying voltage levels. Four Peltier units were connected: two units in series and all four units in parallel configurations. This setup allowed for testing and comparing the refrigerator’s performance under different operational conditions. The result three-section study of the performance of solar-integrated TEC is carried out at different times one hour, for eight hours, and twenty-four hours.

### The MATLAB result

#### The result from MATLAB Simulink of Peltier

**Fig. 6:**
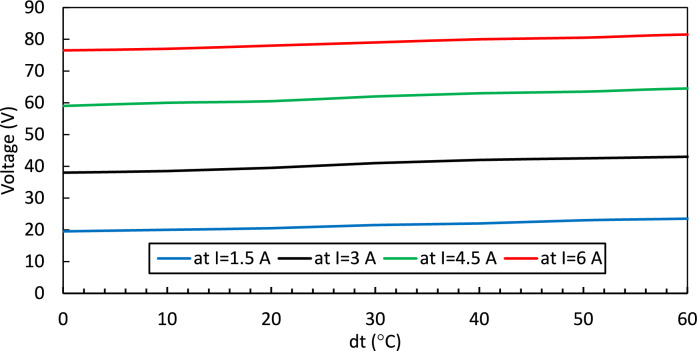
shows the relation between temperature difference and voltage.

Figure 6 illustrates the relationship between voltage and temperature difference at a specific current, and at T_h_ = 25 °C as modeled by the Simulink Peltier model in MATLAB. The main block diagram of the Peltier Module, where the specifications such as ΔT_max_, V_max_, T_c_, and the hot side temperature are entered into the 'Subsystem' block, is provided in the supplementary work (Supplementary file 1). The current, I, and Q_c_ will be varied using the Repeating Sequence Stair block in Simulink according to the user application.

**Fig. 7:**
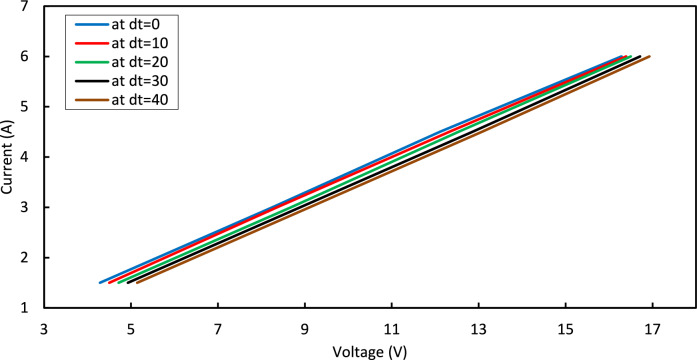
I-V curves for Peltier.

Figure 7 shows the relation between current and voltage at various temperature differences.

(dt = 0.0, 10, 20,30, and 40 °C) and at T_h_ = 25 °C of TEC1-12706.

**Fig. 8:**
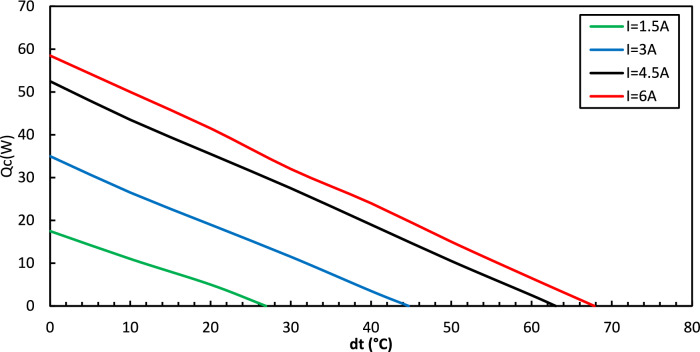
Temperature difference vs. Heat removed for Peltier.

Figure 8 shows the performance curve of Peltier, illustrating the relationship between temperature difference and heat removed at T_h_ = 25 °C for various current values (I = 1.5, 3.0, 4.5, and 6.0 Amperes). From this figure, it is evident that the current varies directly with the heat removed and inversely with the temperature difference.

#### The result from MATLAB Simulink of PV

**Fig. 9:**
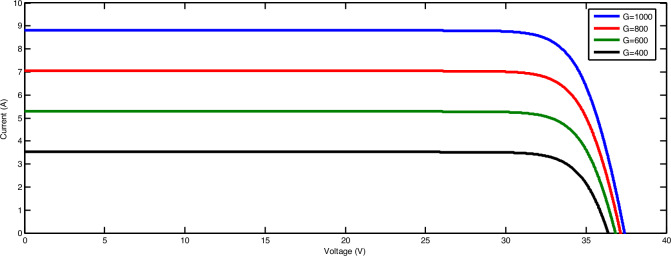
Effect of irradiation on PV current.

**Fig. 10:**
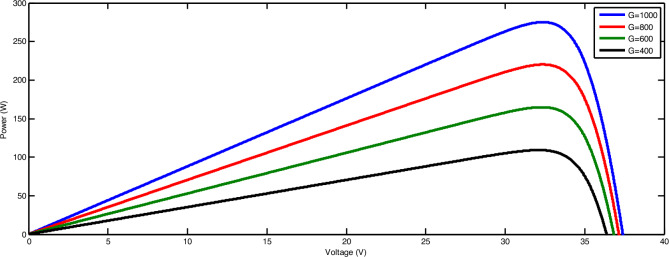
Effect of irradiation on PV power.

The I-V (current–voltage) and P–*V* (power–voltage) curves for different operating radiation levels is shown in Figs. 9 and 10 respectively, The SUNSET PX Series panel was modeled in Simulink using the Electronics library in MATLAB Simulink, depicting various values of solar radiation ranging from 200 to 1000 W/m^2^.

### The result of optimization

**Fig. 11:**
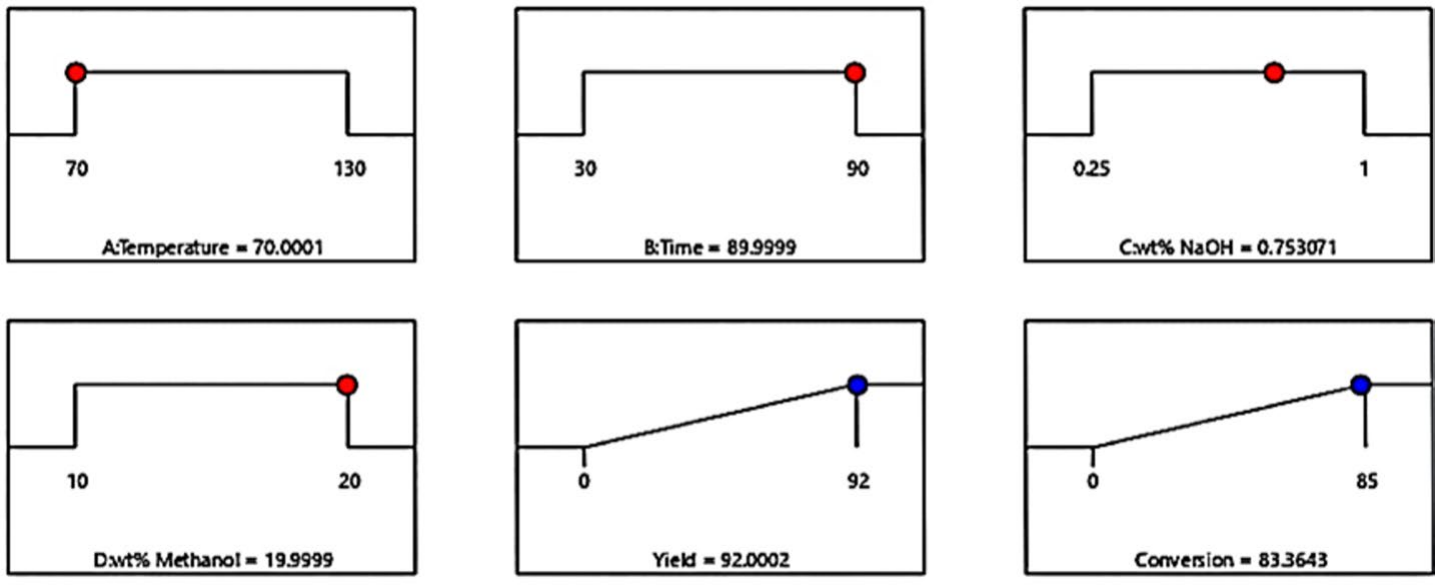
Maximum COP at optimal experimental factors.

The optimization process aimed to obtain the maximum COP under the ranges of the studied operating factors. Maximum COP was achieved from Design Expert software using the numerical optimization type presented in Table 2. The optimal values of the studied factors that give maximum COP was determined. It was found that the maximum COP was 71.089%. These values were achieved at a volt of 4 V, one numbers of Peltier, and a current of 1 A as shown in Fig. 11. The red dots in the figure indicate the optimal input factor values, while the blue colour signifies the maximum outcome value. At volt in range from 4 to 14 V, no. of Peltier units in range from 1 to 4, the response of current in range from 1.006 to 11.7 A, the response of maximum COP%.

#### Coefficient of performance optimization and ANOVA analysis

The coefficient of performance (COP) was evaluated across various experimental runs. Design Expert 13 software was used to generate models that analyze the relationships between process factors and key responses. To assess the quality and significance of these models, an analysis of variance (ANOVA) was conducted at a 96.72% confidence level, focusing on F-values. The quadratic model best represented the COP. However, some terms within these models lacked statistical significance (p-values > 0.1). Therefore, the models were optimized by removing these insignificant terms, resulting in more concise and accurate representations of the process. Equation (5) describes the model obtained by Design Expert software for the COP, and Table 3 summarizes the results of the ANOVA analysis for the COP of the refrigerator box.

**Table 3:** ANOVA Analysis for the response of the only COP.

Source	Sum of Squares	df	Mean Square	F-Value	P-Value	
Model	9203.89	4	2300.97	159.50	< 0.0001	Significant
A (Volt)	2176.87	1	2176.87	150.89	< 0.0001	
B (No. of Peltier)	391.27	1	391.27	27.12	< 0.0001	
AB	195.69	1	195.69	13.57	0.0016	
A^2^	1516.74	1	1516.74	105.14	< 0.0001	
Residual	274.10	19	14.43			
Cor Total	9477.99	23		159.50	< 0.0001	
R^2^ = 0.9711	Adjusted R^2^_=_ 0.9650					

5 $$\text{COPPeltier} +3.44551*\text{ I }+0.799239*{\text{Volt}}^{2}$$

#### Effect of No. of Peltier and voltage on COP%

**Fig. 12:**
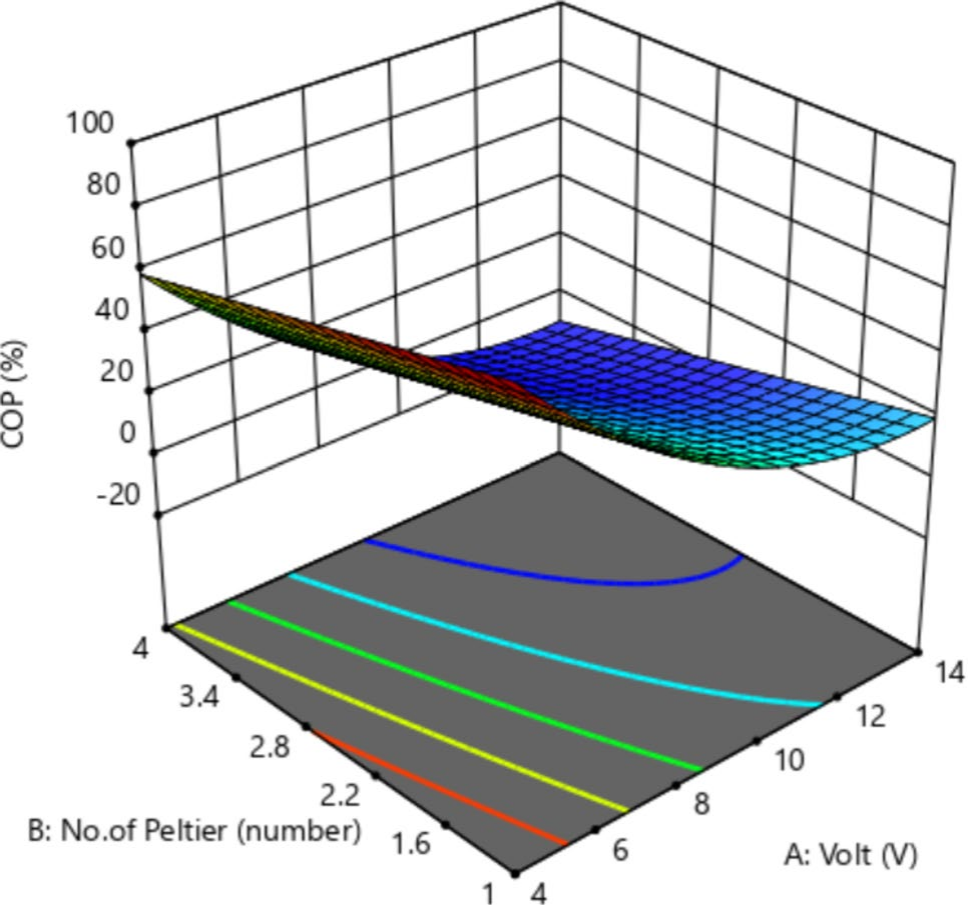
3D response surface plot illustrating interaction effects of No. of Peltier, voltage on the COP.

Figure 12 shows the relationship between voltage, number of Peltier, and COP on a 3D curve. The x-axis represents A: voltage (V) and the y-axis represent B: Peltier axis. The z number represents C: COP. It is noted from the figure that there is an inverse relationship between voltage and COP. This decrease in voltage led to an increase in the COP. It is also clear that the relationship between the number of Peltier and COP is an inverse relationship, as the lower the Peltier number leads to an increase in the COP. Table 4 shows the result for optimization for the COP in the experimental work and calculation by RSM.

**Table 4:** The optimization for the COP.

Type	Volt V	No. of Peltier	Current A	∆T ºC	COP %	Desirability %
Experimental	4	1	1.008	8	77.3	0.978
Calculations	4	1	1	8	71.089	

### Study the performance of the refrigerator during connecting with PV

The outcomes of the experiments are categorized into three main groups: analyzing the refrigerator’s performance for temporary usage for a specific purpose lasting one hour, evaluating its effectiveness in meeting the daily needs of employees during an 8-h work period, and examining its performance throughout the day for 24 h.

#### Study the performance of the refrigerator during the work period for 1 h

**Fig. 13:**
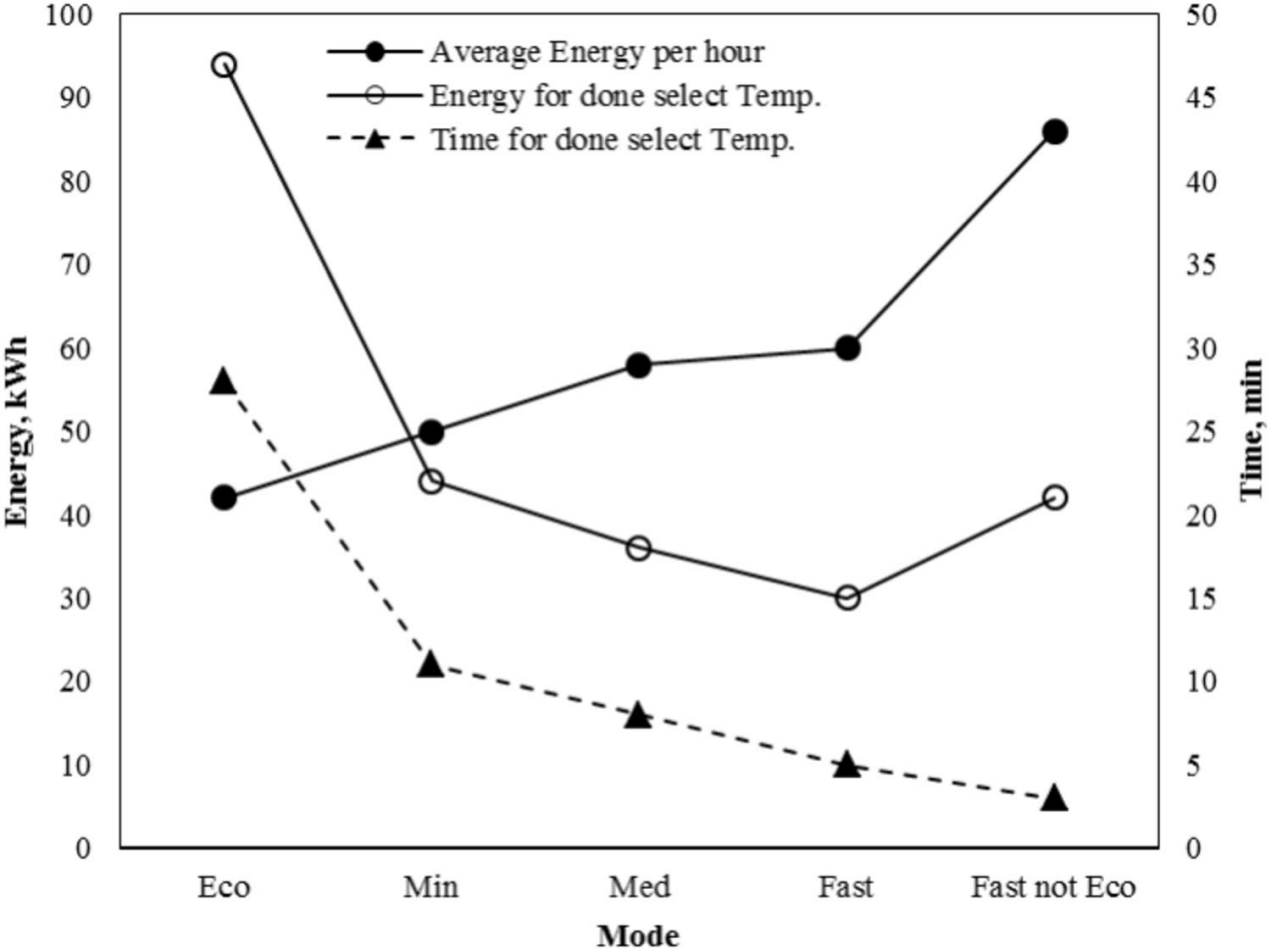
The effect of time on energy at different mode and temperature control 20 ± 0.1 °C.

The first method, working time is one hour. This method without a battery is useful for rapid cooling or using a timer for a specific purpose. Figure 13 shows the five modes: (economic, minimum, medium, fast, and fast not economic). Each of these modes has a specific number of Peltier (1–4) elements and a variable voltage (4–10) volt with control temperatures 20 ± 0.1 °C. The energy consumed in an hour, the energy to reach the cooling temperature, and the time required to reach the cooling temperature were studied for each of the five modes at the specified temperature. Four different control temperatures were studied (20 ± 0.1, 15 ± 0.1, 10 ± 0.1, 5 ± 0.1) °C.

The laboratory experiments, as shown in the first figure, consist of a cold box connected to a solar cell as a power source, accompanied by a control unit and measuring instruments. Several experiments were conducted under certain conditions, such as the ambient temperature, which ranged between 25 to 23 ºC. Five different modes (economic, minimum, medium, fast, and fast not economic), were studied by controlling the temperature of the water inside the cooling box at four different temperatures (20 ± 0.1, 15 ± 0.1, 10 ± 0.1, 5 ± 0.1) °C. Table 5 shows the result of the experimental work. That show the different modes, temperature control range, power per hour (energy, Wh), The energy required to arrive at the temperature-setting point (Wh), Time to reach the specified point (minutes), Min Temp. at setting point (°C).

**Table 5:** Hourly variation of temperature control, energy, and time on an experimental work.

Mode	Temp. control range (°C)	Energy (Wh)	Energy to arrive setting point (Wh)	Time for set point (min)	Min Temp. at setting point(°C)
Eco	20 ± 0.1	40	20	28	19.9
Min	20 ± 0.1	50	11	11	19.9
Med	20 ± 0.1	58	10	8	19.9
Fast	20 ± 0.1	60	10	5	19.9
Fast not Eco	20 ± 0.1	86	18	3	19.9
Eco	15 ± 0.1	42	More than 42	Not arrive at setting point	19
Min	15 ± 0.1	56	More than 56	Not arrive at setting point	16.9
Med	15 ± 0.1	74	30	20	14.9
Fast	15 ± 0.1	78	31	14	14.9
Fast not Eco	15 ± 0.1	135	42	10	14.9
Eco	10 ± 0.1	44	More than 44	Not arrive to setting point	19
Min	10 ± 0.1	58	More than 58	Not arrive to setting point	16.9
Med	10 ± 0.1	85	More than 85	Not arrive to setting point	11
Fast	10 ± 0.1	107	56	29	9.9
Fast not Eco	10 ± 0.1	177	67	17	9.9
Eco	5 ± 0.1	46	More than 46	Not arrive to setting point	19
Min	5 ± 0.1	59	More than 59	Not arrive to setting point	16.9
Med	5 ± 0.1	88	More than 88	Not arrive to setting point	11
Fast	5 ± 0.1	114	More than 114	Not arrive to setting point	6.6
Fast not Eco	5 ± 0.1	228	173	43	4.9

From the table, it is clear that the lowest energy consumption and fastest cooling is the fast not eco mode, as it achieved the shortest cooling time and the lowest energy consumption at different temperatures (20 ± 0.1, 15 ± 0.1, 10 ± 0.1, 5 ± 0.1) °C, and the time to reach the required temperature was (3, 10, 17, 43) min. and energy consumption was (18, 42, 67, 173) Wh respectively.

From Table 5 it is proviso that.

1. The Eco mode uses 20 W until reaching the cooling temperature of 20 ± 0.1 °C within 10 min with total energy during an hour being 40 W.
2. The Med mode uses 30 W energy until reaching the cooling temperature of 15 ± 0.1 °C within 20 min with total energy during an hour was 74 W.
3. The Fast mode uses 56 W until reaching the cooling temperature of 10 ± 0.1 °C within 29 min with total energy during an hour was 107 W.
4. The Fast, not Eco mode uses 107 W until reaching the cooling temperature of 5 ± 0.1 °C within 43 min with total energy during an hour was 228 W.

#### Study the performance of the refrigerator during the work period for 8 h.

**Fig. 14:**
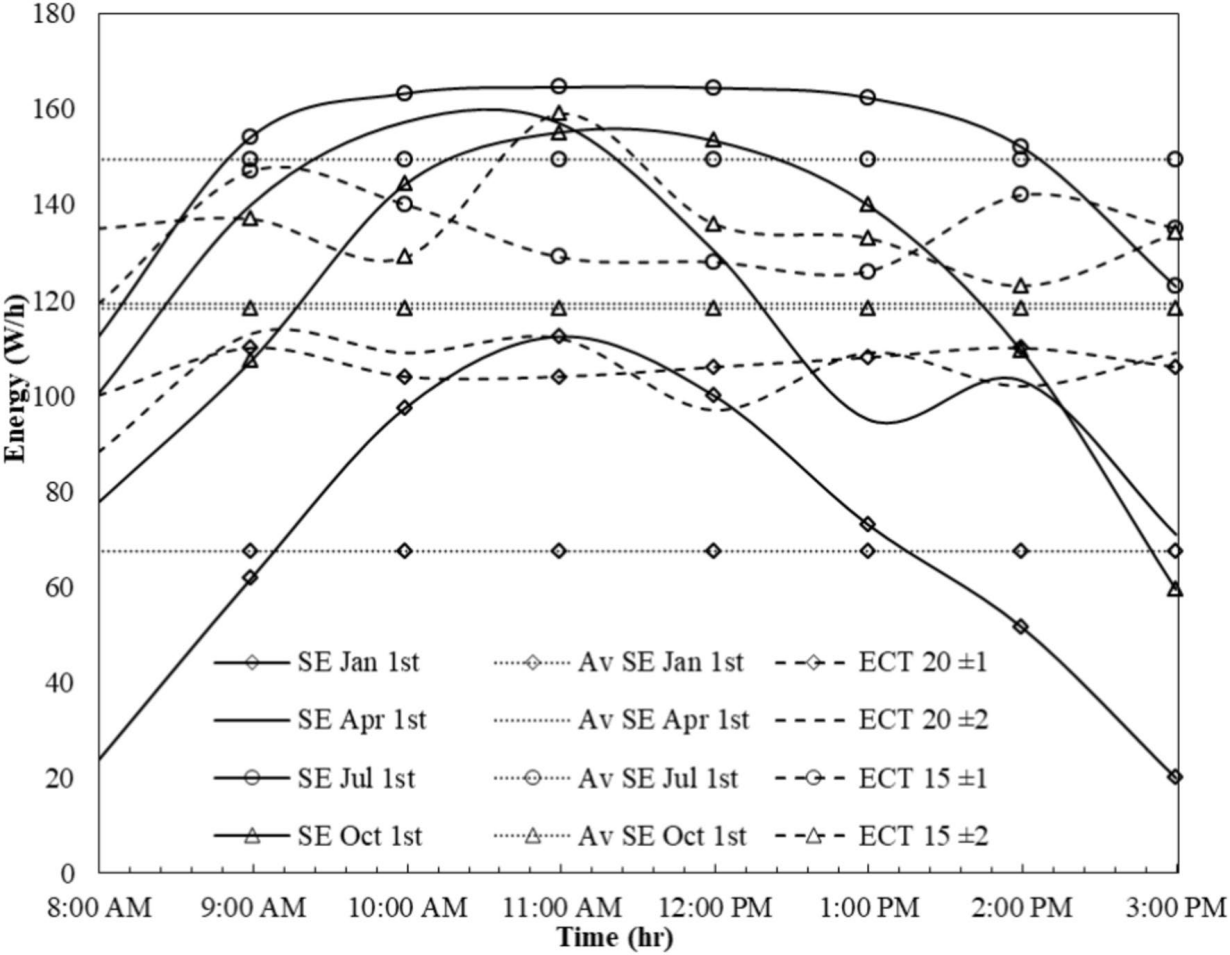
The effect of time on energy throughout 8 h of the day.

In the second method, the system operates without a battery for 8 h, catering to the people who live in remote areas and government workers relying solely on solar radiation. Figure 14 illustrates the measurement of solar energy (SE) across different seasons, with each season represented by a single day: days 1–7 for summer, 1–10 for autumn, 1–1 for winter, and 1–4 for spring. Additionally, it showcases the average solar energy for all four seasons.

During the measurement period from 8 a.m. to 3 p.m., the cooling temperature (ECT) was recorded using the system depicted in Fig. 3. Figure 14 indicates that while spring and fall seasons exhibit similar energy production levels, they differ in their peak times. In spring, the energy peak occurs around 10 a.m., whereas in fall, it happens at 11 a.m.

The average solar energy production across the four seasons was calculated as 149.5, 67.5, 119.3, and 118.3 W-hours for summer, winter, spring, and fall, respectively. This energy production correlates with the refrigerator's needs, varying with the changing seasons. Consequently, energy production increases in summer, coinciding with higher energy consumption. The maximum energy consumption of the refrigerator was recorded as 164.6, 112.39, 157.34, and 155.14 W-hours in summer, winter, spring, and fall, respectively.

#### Study the performance of the refrigerator during the work period for 24 h

**Fig. 15:**
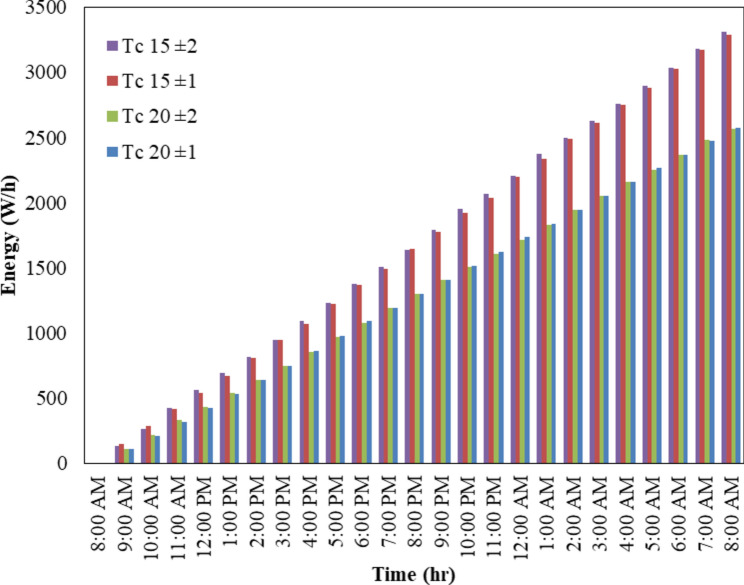
The effect of time on energy throughout the day.

Figure 15 represents the third method that involves continuous operation of the refrigerator throughout the day, representing the total and average energy consumption under different temperature controls. Four Peltier elements are utilized, with two connected in series and then two series pairs connected in parallel. The refrigerator box is connected to a solar system comprising a PV module and a 24 V, 10A battery. The battery ensures continuous operation during night periods only. Temperature control was monitored over 24 h, with on and off cycles implemented for four different temperature settings: 20 ± 1 °C, 15 ± 1 °C, 20 ± 2 °C, and 15 ± 2 °C. The total power consumption for these settings was recorded as 2574, 3289, 2568, and 3314 Wh, respectively. The average power consumption was 107.25, 137.04, 107, and 138.08 W-hours, respectively. Analysis of the results reveals that there is minimal difference in total energy consumption throughout the day when adjusting the temperature control by one or two degrees above or below 20 °C, as well as at 15 °C. However, total energy consumption increases when lowering the cooling temperature from 20 to 15 °C.

Comparing current research on solar PV-driven thermoelectric cooling systems with previous works involves evaluating various aspects such as efficiency, and system design presented in Table 6.

**Table 6:** Comparing current research on solar PV-driven thermoelectric cooling systems with previous works involves evaluating various aspects such as efficiency, and system design.

Authors	Refrigerator type and applications	Methodology	Findings		COP	Variations in temperature
			Power (W)	Cooling (W)		
Present Results by the authors	portable refrigerator based on the Peltier principle for domestic purpose (8 cm × 8 cm × 8 cm)	Using four Peltier at different voltage	4.024	3.11	0.773	The temperature was reduced from 30.9ºC to 4.5ºC in 45 min by using 4 Peltier (0.25 L water)
Biswas et al.^34^	Portable solar thermoelectric cooler for storage of perishable foods	Solar PV system related to the TEC, which consist of TEM, heat sink-fan. The PV and store battery power	53.5	23.8	0.44	The cold side temperature decreased to 5 ± 0.2ºC in 180 min and hot side temperature increased to 40.2 °C
Aboelmaaref et al.^35^	Solar thermoelectric air- conditioner for room air- conditioning (room size: 1.0 m3)	The solar-PV system was integrated with a cooling unit which consists of 16 identical TEMs and 6 rectangular fin heat sinks	2.5 A	30	2.2	The room temperature was reduced to 17ºC. The COP of 0.45 was obtained with thermal and electrical efficiency of 12.06 and 10.27%, respectively
M. Mirmanto et al.^36^	Thermoelectric cooler box with several positions (size: 215* 175* 130) mm^3^	The cooling system of the cooler box comprised a thermoelectric module model TEC1-12706	38.08		0.3	The experiment was conducted for about 18,000 s at the open ambient temperature
Daghigh and Khaledian^37^	Solar thermoelectric cooling-heating system for water cooling	The PV collector was used for thermoelectric cooling system, where 4 TEMs were connected to the water tank	7911 kJ	N/A	5.4	They observed hot and cold side temperatures of TEM were 69 and -3 °C, respectively

## Conclusions

The results showed that the mini refrigerator can be used in many places for economical, fast cooling, and many other systems. Also, can run on solar PV, MATLAB models were built to simulate PV energy and refrigerator cooling temperature. The response surface methodology (RSM) was used for selected optimum COP and mode conditions. During the 8-h operation, the average solar radiation varied across seasons, with values of 149.5, 67.5, 119.3, and 118.3 Wh for summer, winter, spring, and fall, respectively. Concurrently, the average cooling energy consumption was recorded at 107.25, 137.04, 107, and 138.08 Wh for temperature settings of 20 ± 1, 15 ± 1, 20 ± 2, and 15 ± 2 °C. This demonstrates that solar radiation is adequate to generate energy, supporting cooling operations even in summer at temperatures as low as 15 °C, while in spring and fall, temperatures of up to 20 °C suffice.

The "Fast not Eco" mode stands out as the fastest cooling option, ideal for rapid cooling within a short time frame of less than an hour. The Eco mode uses minimum energy until reaching the cooling temperature for a long-time frame of more than an hour.

## Supplementary Information

Supplementary Information.

## Data Availability

The data sets used and/or analyzed during the current study are available from the corresponding author upon reasonable request. All of the data sets used in the study have been either provided or cited in the article.

## Abbreviations

TE: Thermoelectric
TEC: Thermoelectric cooler
TEG: Thermoelectric generator
TU: Thermoelectric unit
COP: Coefficient of performance
Cp: Specific heat of water (J kg ^− ^^1^ K^ − ^^1^)
CFCs: Chlorofluorocarbons
PV: Photo voltaic
Q˙: Heat transfer rate (W)
Tc: The temperature of cold junction (°C)
Th: The temperature of hot junction (°C)
RB: Refrigerator box
TU: Thermoelectric unit
S.S.: Stainless steel
∆T: The cooling temperature difference
PCM: Phase change material
DEC: Direct evaporative cooling

## List of symbols

T_i_: Initial temperature (°C)
C*p*: Specific heat of water (J kg^ − ^^1^ K^ − ^^1^)
I: Electric current (A)
M: Mass of water (kg)
Q˙: Heat transfer rate (W)
Tc: The temperature of cold junction
Th: The temperature of hot junction (°C)
T: Time (s)
W˙: Power consumption (W)
T: Temperature (°C)
V: Voltage (V)
