# Supplementary material for: Optimizing COP by RSM and MATLAB model of mini refrigerator based on thermoelectric units driven by solar photovoltaic
Source: Sci Rep. 2024 Oct 1;14:22766. doi: 10.1038/s41598-024-72500-1 (PMC11445499; doi:10.1038/s41598-024-72500-1)
Supplement: Supplementary file 1 — Supplementary Information. [file 41598_2024_72500_MOESM1_ESM.docx]

**The MATLAB model Code**

Vp = 12;

Th = 44.4;

Tl = 21;

%Tl = 23.8;

%Th = 50.4;

Vr = Vp;

Ir =[ 0 0 0 0];

Q25dt=[0 0 0 0 0];

Q50dt=[0 0 0 0 0];

Qr = [0 0 0 0];

Q25i=0;

Q50i=0;

Ii=0;

Q=0;

%{

Function of (Tl, Th, V)

---------------------------------------------------------------------------

Data

V Data

(Columns)

[V at dt=0 Th=25 V at dt=80 Th=25 V at dt=0 Th=50 V at dt=80 Th=50]

(Lines)

at I = 0, 1.5, 3, 4.5 and 6 A

Q Data

Q25 at Th = 25

Q50 at Th = 50

(Columns)

(Qmax at dt = 0) (Qmin at dt = 80)

Lines are extended to dt = 80 and data is recorded

(Lines)

at I = 0, 1.5, 3, 4.5 and 6 A

Lines of Q at I = 0 are made parallel to lines of I = 1.5A

-------------------------------------------

Steps of solution ... From the first matrix

Inputes

Tl and Th

Output

dt

Inputs

V

Output

Ir(1:2) = I (maximum, minimum) at V and Th = 25

Ir(3:4) = I (maximum, minimum) at V and Th = 50

I25 = I at Th = 25 at run dt

I50 = I at Th = 50 at run dt

-----------------------------------------------

Steps of solution ... From the second and third

Inputes

dt, Qmax and Qmin at Th = 25

dt, Qmax and Qmin at Th = 50

Output

Q25dt at given dt at every I at Th = 25

Q50dt at given dt at every I at Th = 50

Inputes

I

Output

Q25i = output Q at I at Th = 25

Q50i = Output Q at I at Th = 50

Qr matrix is Q0 and Q75 are calculated from Q25 and Q50

Inputs

Th

Output

Q fial output is interpolated from Q data at Qr

%}

% Peltier Data input ------------------------------------------------------

I = [0 1.5 3 4.5 6] % I data

dt = [0 80]

Thd = [0 25 50 75] % Th data

V = [ 0 0 0 0

4 5 4 6

7 9 8 10

11 13 12 14

14 16 16 17]

Q25 = [ 0 -54.4

20 -34.4

30 -27.8

50 -13.6

60 -11]

Q50 = [ 0 -52.8

20 -31.8

40 -22.3

55 -6

65 -2 ]

% Start of Calculation ----------------------------------------------------

% I Calculation -------------

dtr = Th - Tl;

for c = 1 : 4 % Screoll Columns

for v = 1 : 4 % Screoll Lines

if (V(v,c) - Vr)*(V(v+1,c) - Vr)<=0

Ir(c) = I(v) + (Vr - V(v,c))/(V(v+1,c)- V(v,c))* ( I(v+1)-I(v))

break

end

end

end

I25 = Ir(1)+ (dtr - dt(1))/(dt(2)-dt(1))* (Ir(2)-Ir(1))

I50 = Ir(3)+ (dtr - dt(1))/(dt(2)-dt(1))* (Ir(4)-Ir(3))

If = [I25-(I50-I25) I25 I50 I50+(I50-I25)]

for i = 1 : 3 % Screoll Lines

if (Thd(i) - Th) *(Thd(i+1) - Th) <=0

Ii = If(i) + (Th - Thd(i))/(Thd(i+1)- Thd(i))* ( If(i+1)-If(i))

break

end

end

% Ii is the current drawen by the peltier

% Q Calculation ---------------

for i = 1 : 5

Q25dt(i) = Q25(i,1)+(dtr-0)/(80-0)*(Q25(i,2)-Q25(i,1))

Q50dt(i) = Q50(i,1)+(dtr-0)/(80-0)*(Q50(i,2)-Q50(i,1))

end

for i = 1 : 4 % Screoll Lines

if (I(i) - I25) *(I(i+1) - I25) <=0

Q25i = Q25dt(i) + (I25 - I(i))/(I(i+1)- I(i))* ( Q25dt(i+1)-Q25dt(i))

break

end

end

for i = 1 : 4 % Screoll Lines

if (I(i) - I50) *(I(i+1) - I50) <=0

Q50i = Q50dt(i) + (I50 - I(i))/(I(i+1)- I(i))* ( Q50dt(i+1)-Q50dt(i))

break

end

end

Qr = [Q25i-(Q50i-Q25i) Q25i Q50i Q50i+(Q50i-Q25i)]

for i = 1 : 3 % Screoll Lines

if (Thd(i) - Th) *(Thd(i+1) - Th) <=0

Q = Qr(i) + (Th - Thd(i))/(Thd(i+1)- Thd(i))* ( Qr(i+1)-Qr(i))

break

end

end

Ii

if Q <0

Q = 0

end
